# Role of endothelial microvesicles released by *p*-cresol on endothelial dysfunction

**DOI:** 10.1038/s41598-020-67574-6

**Published:** 2020-06-30

**Authors:** Fatima Guerrero, Andres Carmona, Teresa Obrero, Maria Jose Jiménez, Sagrario Soriano, Juan Antonio Moreno, Alejandro Martín-Malo, Pedro Aljama

**Affiliations:** 1 Maimonides Biomedical Research Institute of Cordoba (IMIBIC), Reina Sofia University Hospital, University of Cordoba, Córdoba, Spain; 2 Department of Medicine, University of Cordoba, Córdoba, Spain; 3 Nephrology Unit, Reina Sofia University Hospital, Córdoba, Spain; 4 Spanish Renal Research Network (REDinREN), Institute of Health Carlos III, Madrid, Spain; 5 Department of Cell Biology, Physiology and Immunology, Maimonides Biomedical Research Institute of Cordoba (IMIBIC), University of Cordoba, Córdoba, Spain

**Keywords:** Cell migration, Mechanisms of disease, Senescence, Cardiovascular diseases

## Abstract

Protein bound uremic toxins, such as *p*-cresol, cannot be effectively removed by conventional dialysis techniques and are accumulated in plasma, thus contributing to progression of both chronic kidney disease (CKD) and cardiovascular disease (CVD). Pathological effects of uremic toxins include activation of inflammatory response, endothelial dysfunction and release of endothelial microvesicles. To date, the role of *p*-cresol in endothelial microvesicles formation has not been analyzed. The aim of the present study was evaluate the effects of endothelial microvesicles released by *p*-cresol (PcEMV) on endothelial dysfunction. An in vitro model of endothelial damage mediated by *p*-cresol was proposed to evaluate the functional effect of PcEMV on the endothelial repair process carried out by endothelial cells and microRNA (miRNA) that could be involved in this process. We observed that *p*-cresol induced a greater release of microvesicles in endothelial cells. These microvesicles altered regenerative capacity of endothelial cells, decreasing their capacity for cell migration and their potential to form vascular structures in vitro. Moreover, we observed increased cellular senescence and a deregulation of miRNA-146b-5p and miRNA-223-3p expression in endothelial cells treated with endothelial microvesicles released by *p*-cresol. In summary our data show that microvesicles generated in endothelial cells treated with *p*-cresol (PcEMV) interfere with the endothelial repair process by decreasing the migratory capacity, the ability to form new vessels and increasing the senescence of mature endothelial cells. These alterations could be mediated by the upregulation of miRNA-146b-5p and miRNA-223-3p.

## Introduction

Chronic kidney disease (CKD) is currently a major health problem worldwide^1–3^. Most of the complications associated with CKD involve the cardiovascular system^4^. Thus, cardiovascular diseases (CVD) are recognized as the major cause of mortality in patients with CKD. The underlying mechanisms in CKD are directly related to persistent inflammation, endothelial dysfunction, oxidative stress and vascular calcification^5^. In fact, the chronic microinflammation state present in uremia has been proposed as one of the mechanisms causing endothelial dysfunction, the first step for subsequent development of atherosclerosis.

Uremic toxins, especially protein-bound toxins, are pathogenic agents inducing endothelial dysfunction in CKD^6^. Accumulation of uremic toxins inhibits endothelial proliferation^7^, alter endothelial barrier function^8^, induce apoptosis, cellular senescence and endothelial damage^9,10^ and promote endothelial vesiculation^11^. *p*-cresol, one of the major protein-bound uremic toxins, is accumulated in the serum of CKD patients, thus contributing to the maintenance of this chronic inflammatory state^12^. *p*-cresol has been associated with overall and cardiovascular mortality^13^, and progression of CKD^14^. In vitro evidences suggest a deleterious effect of *p*-cresol on the endothelium^7,8^.

Endothelial microvesicles (EMV) (also called endothelial microparticles) have been recently described as new markers of endothelial dysfunction. EMV are membrane vesicles (0.1–1 μm diameter) that are actively released from cells in response to different stimuli, including inflammation, oxidative stress, etc.^15^. A number of studies have shown that uremia is associated with increased numbers of circulating EMV^11,16^. Furthermore, circulating EMV has been associated with impaired vascular function in hemodialysis patients^16^.

Different studies have shown that some uremic toxins, such as indoxyl sulfate and *p*-cresol, induce the formation of EMV in cultured endothelial cells^11,17^. In a previous study, we observed that microvesicles derived from indoxyl sulfate-treated endothelial cells impaired endothelial progenitor cells-reparative process^17^. Nevertheless, little is known about the direct effect of EMV released in response to uremic toxins on vascular homeostasis, specially in mature endothelial cells. Thus, the objective of this study was to analyze the effect of EMV derived from *p*-cresol-treated human umbilical vein endothelial cells (HUVECs) on different processes related to endothelial damage, such as senescence, angiogenesis, and migration in mature endothelial cells.

## Results

### *p*-cresol induced increased EMV release in HUVECs

**Figure 1:**
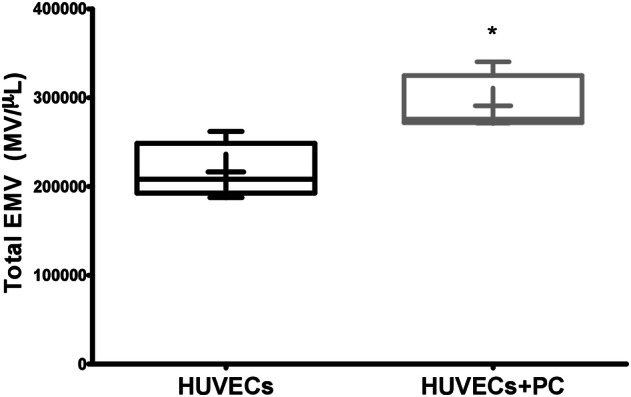
Human umbilical vein endothelial cells (HUVECs)-derived microvesicles (EMV) assessed by flow cytometry. Absolute number of EMV per microliter in PC-treated (HUVECs + PC) and untreated HUVECs. Results are the means ± SD of six independent experiments. *p < 0.01 versus untreated HUVECs.

Since EMV are associated with endothelial activation, we first determined the effect of *p*-cresol on EMV formation in HUVECs. We observed elevated EMV release in *p*-cresol-treated HUVECs (MV/µl) with respect to control cells (290,830 ± 30,684 vs. 216,430 ± 29,535; *p* = 0.0002) (Fig. 1).

### *p*-cresol derived EMV induce senescence in mature endothelial cells

**Figure 2:**
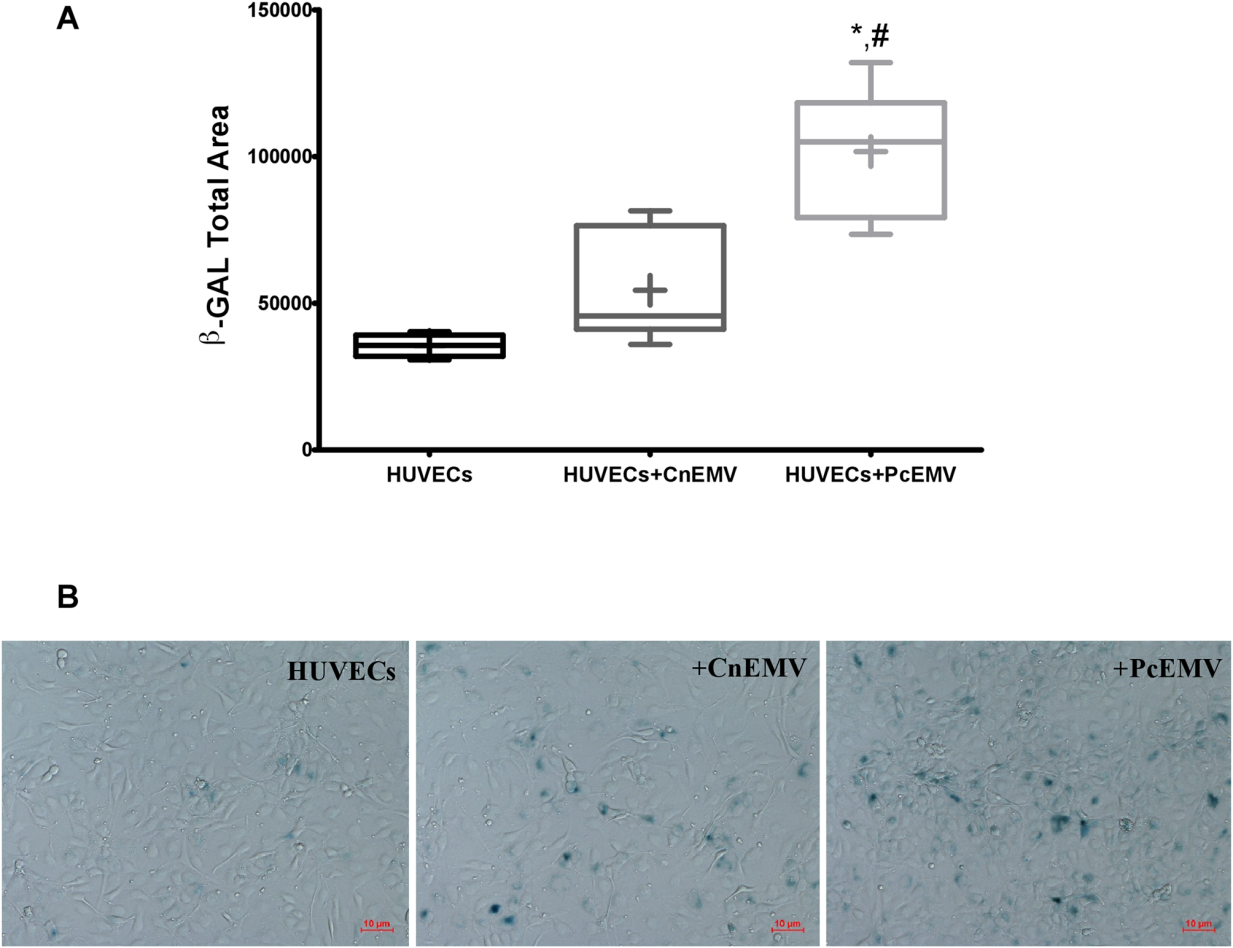
EMV derived from *p*-cresol treated-HUVECs induces senescence in human umbilical vein endothelial cells. Senescent-associated β-Galactosidase activity staining. (**A**) Stained levels of β-Galactosidase in HUVECs across all experimental conditions. (**B**) Representative images of inverted optical microscopy of the senescence studies of control HUVECs, HUVECs treated with CnEMV and PcEMV. Data are the means ± SD of six independent experiments. *p < 0.01 versus HUVECs; ^#^*p* < 0.01 versus HUVECs + CnEMV.

To investigate the role of *p*-cresol derived EMV (PcEMV) on senescence, β-galactosidase (β-Gal) staining was carried out in cultured HUVECs. As shown in Fig. 2, the number of β-Gal labeled pixels in HUVECs treated with PcEMV was significantly greater than the number of labeled pixels in all other groups. No significant differences were observed between CnEMV-treated HUVECs and control ones.

### *p*-cresol derived EMV inhibit endothelial cell migration

**Figure 3:**
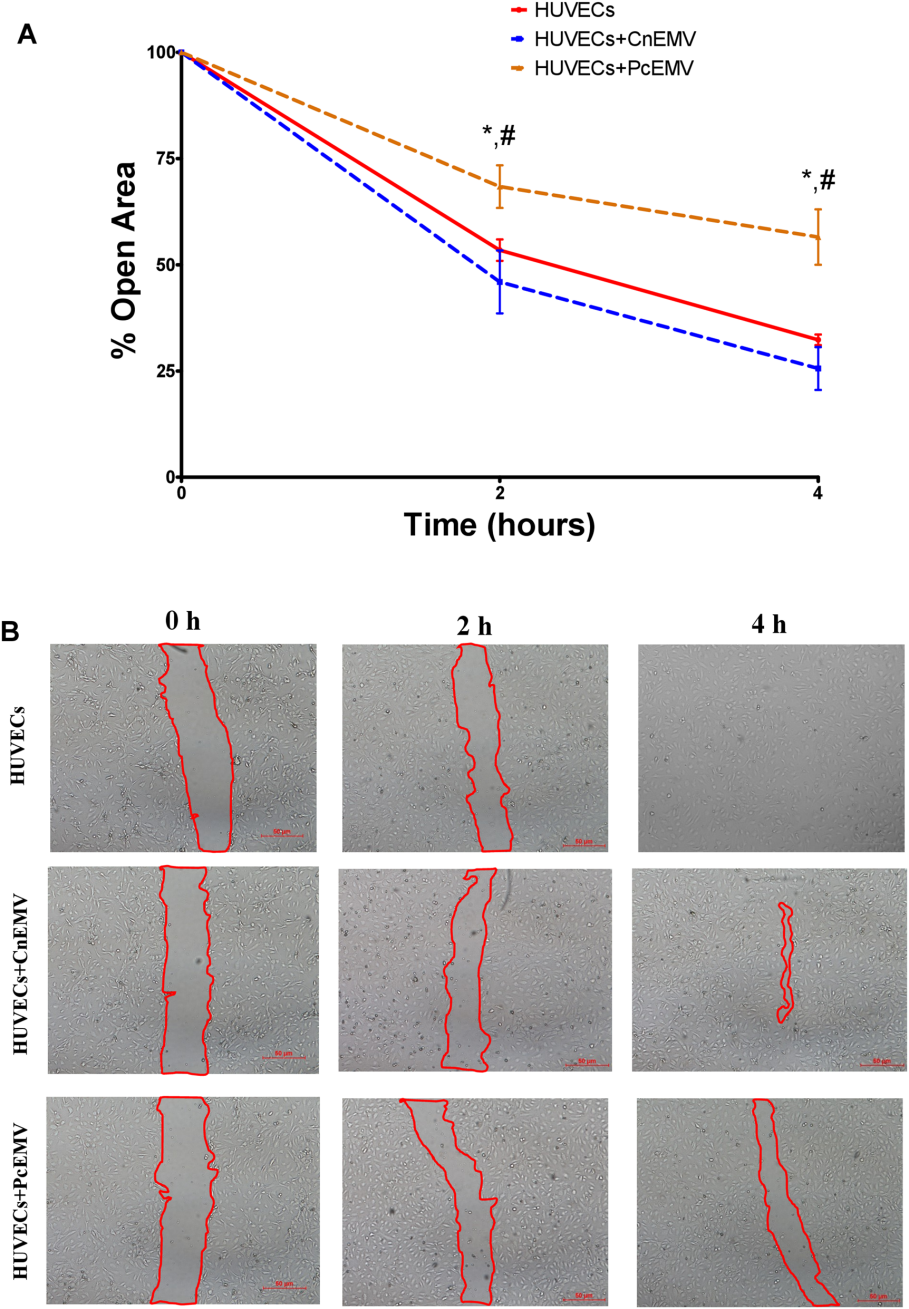
Wound healing in HUVECs monolayers. Time course of changes in the size of the remaining wound. (**A**) The data points represent the % open area. (**B**) Representative images of inverted optical microscopy of the wound test in HUVECs under the indicated conditions (extent of the wound marked with red lines). Results are the means ± SD of six independent experiments. *p < 0.05 versus HUVEC; ^#^*p* < 0.05 versus HUVECs + CnEMV.

To determine the effect of *p*-cresol derived EMV on HUVECs migration, we performed the wound healing assay at different time points (Fig. 3). Two hours after the wound was performed, we observed a significant decrease in the size of the wound in control and CnEMV-treated HUVECs with respect to those stimulated with PcEMV (*p* = 0.035 for both conditions). This fact was more accentuated 4 h later. Hence, the treatment with PcEMV reduced migration in mature endothelial cells.

### Anti-angiogenic effect of PcEMV on HUVECs

**Figure 4:**
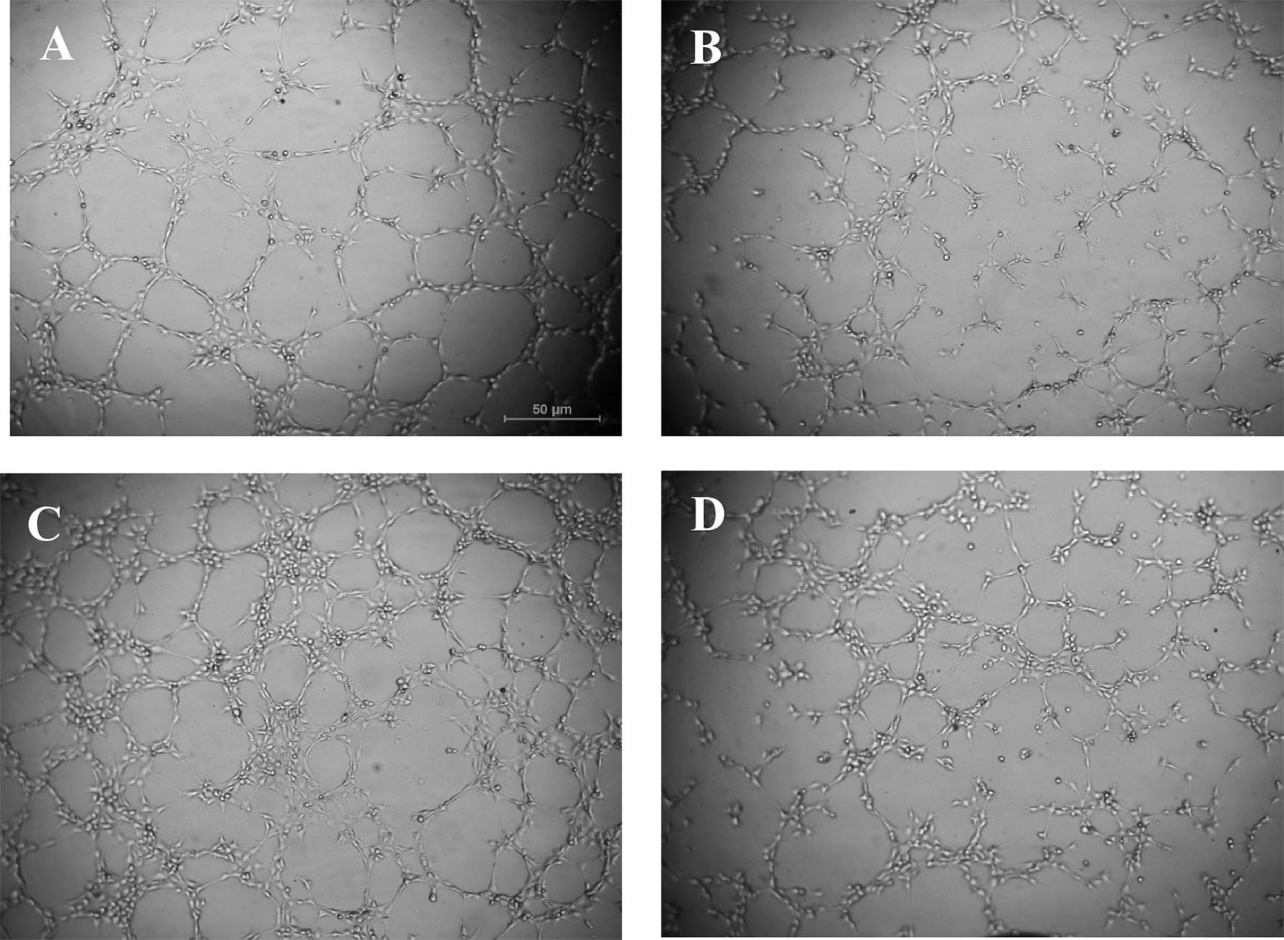
Optical microscopy representative images of the formation of vessels in the in vitro angiogenesis assay on the semi-natural matrix, Matrigel, 2 h after seeding. Representative image of (**A**) HUVECs treated with endothelial basal medium (EBM) with 10% fetal bovine serum (FBS) used as a positive control, HUVECs without EMV (**B**) and HUVECs treated with CnEMV (**C**) or PcEMV (**D**).

We next evaluated the capacity of EMV to modulate angiogenesis in mature endothelial cells*.* As shown in Fig. 4, PcEMV-treated HUVECs had a lower capacity to form new vessels in the 3D matrix. In agreement with this observation, we also observed a reduced number of Nb master junctions, decreased number Nb segments and a lower total length of vessels in those HUVECs treated with PcEMV as compared with CnEMV (Table 1).

**Table 1:** Angiogenic parameters.

Treatment	NB master junctions	Nb segments	Nb branches	Total length
HUVECs +	66.0 ± 10.6	183.7 ± 24.2	80.2 ± 5.7	15,570.7 ± 869.4
HUVECs −	47.5 ± 5.4	128.7 ± 10.6	63.0 ± 1.3	13,908.7 ± 496.1
HUVECs + CnEMV	86.5 ± 5.9*^#^	234.7 ± 15.8*^#^	64.6 ± 1.5	17,157.3 ± 480.8*^#^
HUVECs + PcEMV	58.9 ± 5.3	150.2 ± 13.8	63.7 ± 1.9	14,509.6 ± 766.0

Tube formation was evaluated by measurement of Nb master junctions, Nb segments, Nb branches, and total length after treatment with microvesicles derived from HUVECs. HUVECs with 10% FBS (HUVECs +), HUVECs without EMV (HUVECs −), and HUVECs treated with CnEMV (HUVECs + CnEMV) or PcEMV (HUVECs + PcEMV). Data are the means ± SEM of six independent experiments. *p < 0.05 versus HUVECs; ^#^*p* < 0.05 versus HUVECs + PcEMV.

### microRNA-146b-5p and microRNA-223-3p are increased in PcEMV-treated HUVECs

**Figure 5:**
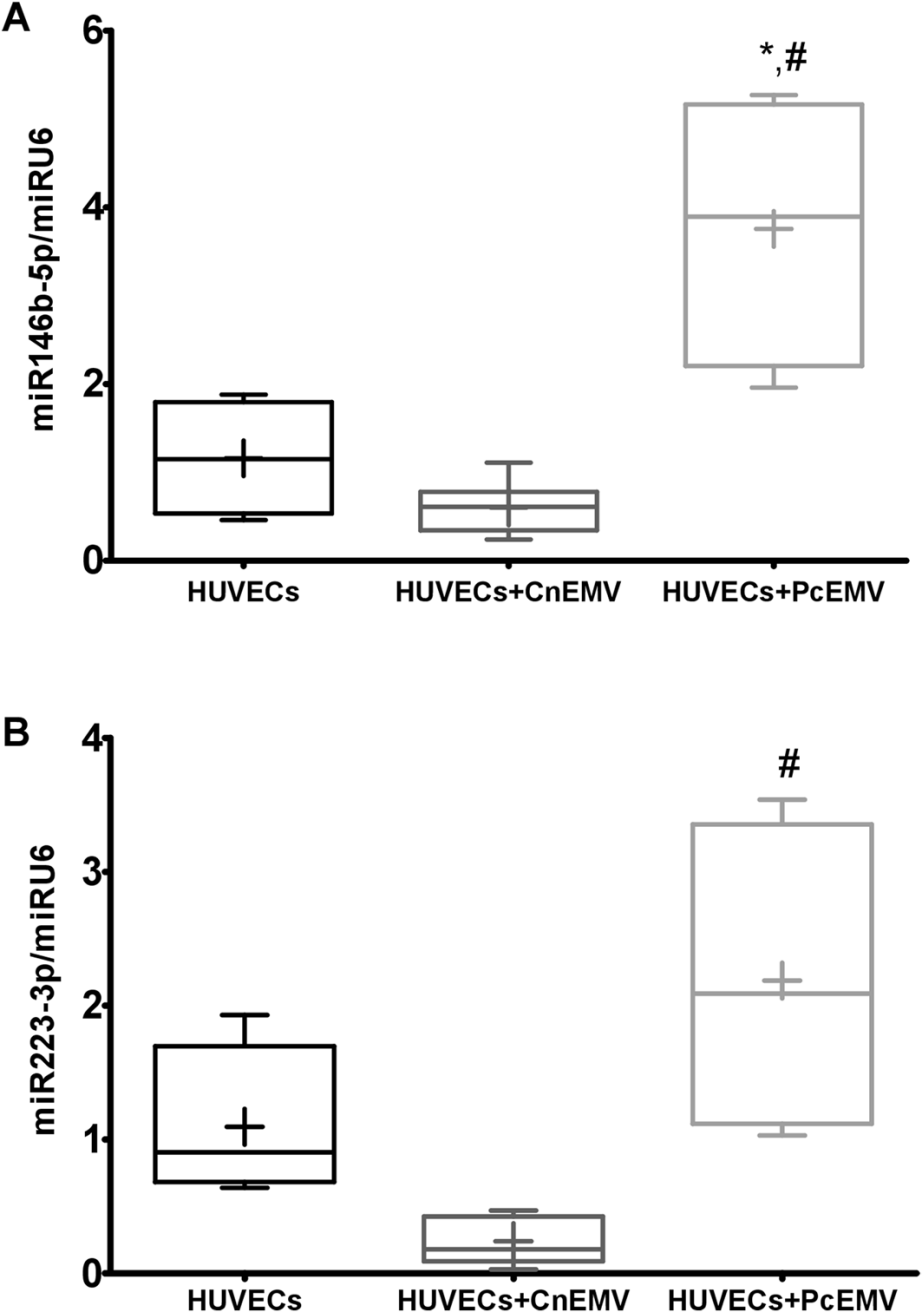
Relative expression of miR-146b-5p and miR-223-3p in HUVECs stimulated with EMV. Data are means ± SD of six independent experiments. *p < 0.01 versus HUVECs; ^#^*p* < 0.01 versus HUVECs + CnEMV.

On the other hand, miRNAs are highly expressed in endothelial cells, and recent data suggest that they regulate important aspects of vascular function. As shown in Fig. 5, addition of CnEMV to cultured mature endothelial cells resulted in a markedly reduced expression of miRNA-146b-5p. However, when HUVECs were cultured with PcEMV, the expression of miRNA-146b-5p was significantly increased compared to the other groups (*p* < 0.001).

Similarly, by adding PcEMV we observed an increase in miRNA-223-3p expression levels with respect to CnEMV (*p* = 0.008). We did not observe significant differences in the other study groups.

## Discussion

In CKD patients, vascular endothelium is in continuous contact with uremic toxins accumulated in the bloodstream. *p*-cresol accumulation causes endothelial dysfunction^18^, and further CKD progression and increased CVD risk^19,20^. *p*-cresol is a protein bound uremic toxin that is difficult to remove by conventional dialysis techniques. In our in vitro study, we have used the *p*-cresol concentration observed in uremic patients on hemodialysis^21^ to simulate the uremic status of CKD patients. We demonstrated that *p*-cresol acts directly on endothelial cells by increasing EMV release and impairing endothelial reparative process.

In response to *p*-cresol, endothelial cells release EMV, as previously reported^22^. In agreement with this observation, other authors have observed an increase in circulating EMV levels in different pathologies associated with endothelial dysfunction, such as antiphospholipid syndrome^23^, CVD^24^ and CKD on hemodialysis^11^; and it has been suggested that this increased endothelial vesiculation could be indicative of endothelial dysfunction due to uremia. Our research group previously observed that in uremic conditions, endothelial cells release an increased amount of EMV, which may be involved in endothelial dysfunction^17^. Other authors have also described that uremic toxins are capable of producing vesicle endothelial cells and can induce endothelial dysfunction in vitro^9,10^. In accordance with these studies, we observed an increased release of EMV after addition of *p*-cresol.

In CKD patients, uremic toxins, inflammatory cytokines, and other stress-related factors induces premature endothelial senescence^25^. There are few works that relate microvesicles to the process of cellular senescence, although it is well known that senescent cells produce more EMV^26^. Our findings show that EMV derived from *p*-cresol treatment increase the number of senescent endothelial cells.

In recent years there are a large number of studies that have reported a key role of EMV in angiogenesis^27^ and endothelial repair^28^. The endothelial repair process involves the recruitment of endothelial progenitor cells (EPCs) from the bone marrow. There is a close relationship between the reparative functions of EPCs and EMV^29^. Interestingly, CKD patients showed increased release of EMV and decreased number of circulating EPCs and, as consequence, and impairment of the reparative mechanism of endothelium^30,31^. We have recently described that, EPCs treated with EMV from HUVECs exposed to indoxyl sulfate reduced their ability to form colony forming units and their angiogenic capacity^17^. In this work we have studied the autocrine role of EMV on endothelial reparative processes, observing that *p*-cresol treated HUVECs released PcEMV, resulting in an inhibition of in vitro angiogenesis. According to our results, other authors have also postulated that high levels of uremic toxins in the blood of CKD patients may disturb angiogenesis^32^. Taken together, our results suggest that PcEMV may amplify endothelial damage induced by *p*-cresol.

On the other hand, previous studies have reported that *p*-cresol stimulate the vascular endothelium and inhibited endothelial proliferation and repair, which might be an important mechanism of accelerated atherosclerosis^7,33^. In the present study, we have observed that PcEMV exhibited a deleterious effect on HUVECs proliferation decreasing its capacity to repair the endothelium. We must add that endothelial cells from uremic patients are chronically exposed to uremic toxins, so one can suppose that these effects may be even more deleterious in patients with CKD.

As a result, in order to elucidate the mechanisms underlying the effects of EMV, we analyzed the expression of miRNAs related to cardiovascular disease, such as miRNA-146b-5p and miRNA-223-3p. The results of our study showed that dysfunctional HUVECs treated with PcEMV showed increased levels of miRNA-146b-5p and miRNA-233-3p. miRNAs are highly expressed in endothelial cells, and recent data suggest that they regulate important aspects of vascular function^34^. In fact, it is known that miRNAs are involved in the regulation of angiogenesis and endothelial function^35,36^.

miRNA-146b-5p has been reported in inflammatory disorders such as rheumatoid arthritis, systemic lupus erythematosus and psoriasis^37^. An earlier study revealed upregulation of miRNA-146b-5p in peripheral blood mononuclear cells in CKD patients on hemodialysis^38^. Whereas miRNA-146 has been strongly studied in monocytes and macrophages, it is not well known the role of miRNA-146 in endothelial cells. Pfeiffer D et al. have showed an elevated expression of miRNA-146b after stimulation of endothelial cells with supernatant of LPS-treated THP-1 monocytes, which results in an increased in the expression of pro-inflammatory cytokines, such as tumor necrosis factor -alpha (TNF-α), interleukin-6 (IL-6) and interleukin-8 (IL-8)^39^. On the other hand, miRNA-146 has been associated with cellular senescence and an increase in angiogenic activity, as a regulatory mechanism of inflammation^40,41^. Other authors observed that miRNA-146b-5p silencing inhibits proliferation and cell migration in vascular smooth muscle cells in response to platelet-derived growth factor (PDGF)^42^. Recently, miRNA-146b-5p has been identified as a new potential angiogenesis regulatory factor since miRNA overexpression decreases migration and tube formation activity of colony-forming endothelial cells from patients with coronary heart disease^43^. In our study, we found that increased expression levels of the miRNA-146b-5p and miRNA-223-3p were linked with senescence and reduced proliferative and angiogenic capacity of mature endothelial cells treated with PcEMV.

miRNA-223-3p is another important miRNA associated with endothelial dysfunction and vascular calcification^44–46^. miRNA-223-3p deregulation has been associated with CKD progression, inflammation and kidney dysfunction^47^. Indeed, miRNA-223-3p is a well-known miRNA related to inflammation as well as with anti-angiogenic and pro-apoptotic properties^32,48,49^. Moreover, a recent study, reported that miRNA-223-3p is also associated with HUVECs angiogenesis inhibition, increased endothelial apoptosis, and enhanced vascular smooth muscle cells calcification^37^. Therefore, our combined results are in favor of a direct relationship between miRNA deregulation and vascular disease in the uremic patient. Further studies are required to identify and validate the specific role of miRNAs on endothelial dysfunction in uremia.

## Conclusion

In summary, *p*-cresol promotes EMV release causing dysregulation of vascular homeostasis. The microvesicles generated in endothelial cells treated with *p*-cresol (PcEMV) interfere with the endothelial repair process by decreasing the migratory capacity, the ability to form new vessels and increasing the senescence of mature endothelial cells. These alterations could be mediated by the upregulation of miRNA-146b-5p and miRNA-223-3p. All these data show that microvesicles and miRNAs might be a biomarker of endothelial damage and should probably be considered as therapeutic targets to prevent endothelial damage and vascular disease in CKD.

## Materials and methods

### Cell culture and reagents

HUVECs were obtained from Cell Systems (Clonetics, Solingen, Germany) and cultured at 37 °C in a 5% CO_2_ atmosphere and 95% humidity in endothelial cell basal medium (EBM) plus endothelial cell-growth medium supplements (EGM, Cambrex Bioscience, Walkersville, MD) and 10% fetal bovine serum (FBS) (Invitrogen, Carlsbad, CA, USA). Cells were detached from culture surface by trypsin (Lonza, Clonetics, CC-5012) whenever necessary for maintenance, expansion and required experiments. HUVECs were used for experiments between passages four and nine. HUVECs at 80% confluence were incubated with or without *p*-cresol at 25 μg/ml for 24 h at 2% FBS to keep the cell in quiescence state. We first established the experimental model using a concentration- and time- response curve (see Data Supplementary Fig. 1).

For studies with HUVECs, cells were grown in 6-well plates (ref: 140,675, Thermo Scientific). When the cells in culture reached 80% confluence, the treatment was started. Three experimental groups were studied: one group of EMV-free cells (HUVECs) and two groups of cells treated with EMV (at a concentration of 10^5^ (EMV/ml)) obtained from the culture medium of *p*-cresol -treated and untreated HUVECs. The cultures were kept in an oven at 37 °C, in a humid atmosphere and 5% CO_2_ (Heracell 150, Heraeus), with 2% FBS culture medium for 5 days. The treatment was renewed every 2 days.

### Endothelial microvesicles isolation and quantification

As previously our group has described^17^, EMV from the culture medium of *p*-cresol -treated and untreated HUVECs were isolated by ultracentrifugation. The media was centrifuged (Heraeus Labofuge 400R) at 409×*g* for 5 min at 4 °C to remove any intact cells, followed by centrifugation at 789×*g* for 10 min at 4 °C to remove cell debris. The media was then transferred to ultracentrifuge 25 × 89 mm polypropylene tubes (Beckman Coulter, Brea, CA, USA) and centrifuged at 18,000×*g* for 90 min at 10 °C in an Optima XPN-100 ultracentrifuge with a 70Ti rotor (Beckman Coulter). The supernatant containing EMV-free media was removed and the pellets containing EMV were resuspended in PBS sterile and quantified by flow cytometry (FC500 Series, Beckman Coulter). Absolute values of EMV were calculated using the following formula: (EMV counted × standard beads/L)/ standard beads counted (Flow-Count Fluorospheres, Beckman Coulter). Results were expressed as the number of EMV per microliter of culture medium. EMV derived from untreated HUVECs were defined as control EMV (CnEMV) and *p*-cresol-treated HUVECs were defined as *p*-cresol EMV (PcEMV).

### Senescence assay

Senescent cells were identified by using the Senescence-Galactosidase Staining Kit (MBL International Corporation, Catalog #JM-K320-250) according to the manufacturer’s protocol. Briefly, cells were washed twice with 2 ml phosphate buffered saline (PBS) (1X) and resuspended in 2 ml of fixing solution for 15 min at room temperature. Then, the cells were washed twice with PBS (1X), the staining solution was added and allowed to incubate overnight at 37 °C without CO_2_. Afterward, senescence-associated beta-galactosidase-positive cells (senescent cells) were identified as blue-stained cells under light microscopy (20X) (OPTIKA Microscopes, Ponteranica, BG, Italy) and quantified with the ImageJ analysis software (https://rsb.info.nih.gov/ij/).

### Wound healing assay

The wound assay aims to study cell migration. In each well of the culture plate, the monolayer was scratched across the centre using a sterile micropipette tip. Subsequently, the cells were then rinsed with PBS to discard the cellular debris detached from the "wound" zone. Next, culture medium was added and HUVECs were incubated at 37 °C and 5% CO_2_. The wound pictures were captured by optical microscopy (OPTIKA Microscopes) the same positions at 0, 2 and 4 h, after which wound area was determined using ImageJ software.

### Angiogenesis assay in matrigel

To evaluate the potential of HUVECs to form vascular structures, an in vitro angiogenesis test was performed on Matrigel substrate using the Cell Biolabs Endothelial Tube Formation test kit (Cell Biolabs, San Diego, CA, USA). Ninety-six-well sterile plate (Corning Costar, Cambridge, MA) were coated with 50 μl gel solution at 4ºC and incubated at 37 °C and 5% CO_2_ for 30 min. A total of 2.5 × 10^3^ cells/well were seeded for the angiogenesis assay. In parallel, 10% FBS was administered as an internal positive control. After 2 h, photographs were taken with an optical inverted microscope (OPTIKA Microscopes, Ponteranica, BG, Italy). Length of tube-like structures per field was quantified with “angiogenesis analyzer” and an automated analysis was performed with the ImageJ software. Results were expressed as previously described by Izuta et al.^50^.

### RNA isolation

Total RNA from stimulated HUVECs was extracted with TRI-Reagent (Sigma-Aldrich, MO, USA) and quantified by spectrophotometry (NanoDrop ND-1000 UV–Vis Spectrophotometer; NanoDrop Technologies, Wilmington, DE, EE.UU).

### RTqPCR and miRNA expression

For quantification of mature miRNA levels, trizol purified RNA was used. cDNA was synthesized from 100 ng RNA using miRCury LNA RT-Kit (Qiagen) according to the manufacturer’s protocol (PCR System 9,700, GeneAmp, PE Applied Biosystems). Each cDNA was amplified using the miRCury LNA SYBR Green Kit (Qiagen). The reaction mix was first incubated at 95 °C for 2 min. Then, 40 cycles of 95 °C for 10 s and 56 °C for 1 min following by 95 °C for 1 s, 60 °C for 15 s and 95 °C for 1 s. Finally, 40 °C for 30 s. Assay ID used were: hsa-miR-146b-5p (204,553, Exiqon), hsa-miR-223-3p (YP00205986CC, Qiagen) and U6 snRNA (YP00203907CC, Qiagen). The expression level of these genes was evaluated by quantitative RT-PCR (Light cycler 96, Roche Diagnostics, Basel, Switzerland). The relative amount of each miRNA was calculated using the comparative threshold (Ct) method with ΔCt, Ct (miRNA)–Ct (U6 snRNA) in mature endothelial cells.

### Statistical analysis

Data are represented as means ± SEM or means ± SD of six independent experiments. The data distribution was performed with the Shapiro–Wilk test to verify the normal distribution. Comparisons between paired or unpaired data were analyzed by T-Student's-test. The analysis of variance ANOVA with Bonferroni correction for multiple comparisons was used. Non-parametric data were analyzed by the Mann–Whitney U-test or Kruskal–Wallis test. *P* value < 0.05 was considered statistically significant. Data were analyzed using SPSS Statistics software version 25.0 (SPSS, Inc., Chicago, Ill, United States) or the GraphPad Prism 6.0c (GraphPad Software, La Jolla, CA).

## Supplementary information

Supplementary information
