# Supplementary material for: Role of endothelial microvesicles released by p-cresol on endothelial dysfunction
Source: Sci Rep. 2020 Jun 30;10:10657. doi: 10.1038/s41598-020-67574-6 (PMC7326964; doi:10.1038/s41598-020-67574-6)
Supplement: Supplementary file 1 — Supplementary information [file 41598_2020_67574_MOESM1_ESM.pdf]

Role of endothelial microvesicles released by p-cresol on endothelial dysfunction.

Autores: Fatima Guerrero [1,2,†,*]; Andres Carmona [1,†]; Teresa Obrero [1]; Maria Jose Jiménez [1]; Sagrario Soriano [1,3,4]; Juan Antonio Moreno [1,5,*]; Alejandro Martín-Malo [1,2,3,4,‡]; Pedro Aljama [1,2,‡].

1 Maimonides Biomedical Research Institute of Cordoba (IMIBIC), Reina Sofia University Hospital, University of Cordoba, Cordoba, Spain.
2 Department of Medicine, University of Cordoba, Cordoba, Spain.
3 Nephrology Unit, Reina Sofia University Hospital, Cordoba, Spain.
4 Spanish Renal Research Network (REDinREN), Institute of Health Carlos III. Madrid, Spain.
5 Department of Cell Biology, Physiology and Immunology, University of Cordoba, Cordoba, Spain.
† These authors contributed equally to this paper.
‡ These authors share senior authorship.

*Correspondence and reprint requests: Fátima Guerrero, PhD. Maimonides Biomedical Research Institute of Cordoba (IMIBIC). Email: fatima.guerrero@imibic.org; and Juan Antonio Moreno, PhD. Department of Cell Biology, Physiology and Immunology, Maimonides Biomedical Research Institute of Cordoba (IMIBIC), University of Cordoba, Spain. Email: juan.moreno@uco.es

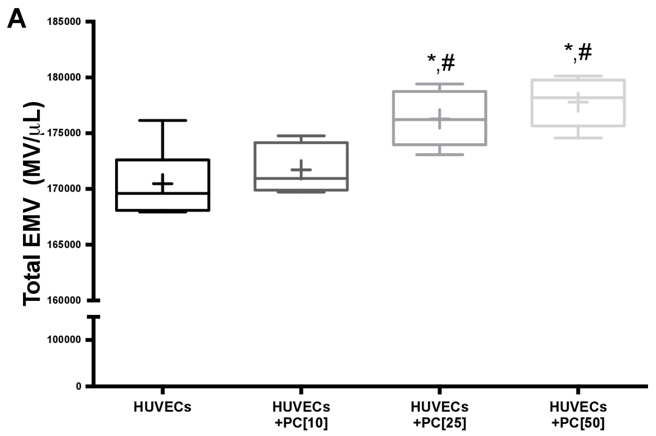

A
Total EMV (MV/μL)
185000
180000
175000
170000
165000
160000
100000
0
*,#
*,#
HUVECs
HUVECs +PC[10]
HUVECs +PC[25]
HUVECs +PC[50]

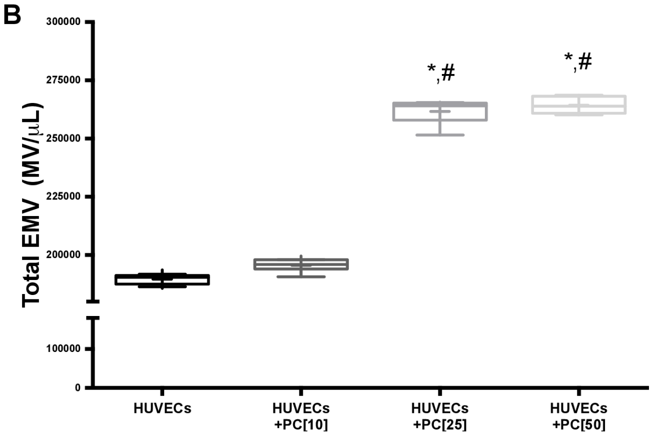

B
Total EMV (MV/μL)
300000
275000
250000
225000
200000
100000
0
*,#
*,#
HUVECs
HUVECs +PC[10]
HUVECs +PC[25]
HUVECs +PC[50]

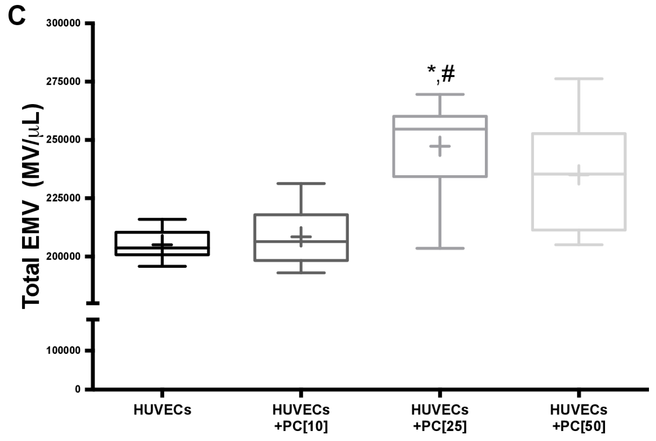

C
Total EMV (MV/μL)
300000
275000
250000
225000
200000
100000
0
*,#
HUVECs
HUVECs +PC[10]
HUVECs +PC[25]
HUVECs +PC[50]

Figure Legend

Data Supplementary Figure 1. Dose- and time-dependent effects of p-cresol on EMV release by HUVECs. HUVECs were treated with different concentrations of p-cresol (PC) to determine the production of EMV per microliter at 12 h (A), 24 h (B) and 48 h (C). Results are expressed as mean ± SD; * $p< 0.05$ vs HUVECs, # $p< 0.05$ vs HUVECs+PC[10].
